# Komplikationen von Laser und Energie‐basierten Systemen in der Dermatologie: Klassifikation, Management und Prävention

**DOI:** 10.1111/ddg.70102x

**Published:** 2026-03-09

**Authors:** Lynhda Nguyen, Wolfgang Kimmig, Stefan Hammes, Stefan W. Schneider, Nikolaus Seeber

**Affiliations:** ^1^ Klinik und Poliklinik für Dermatologie und Venerologie Universitätsklinikum Hamburg‐Eppendorf, Hamburg; ^2^ Klinik und Poliklinik für Mund‐ Kiefer‐ und Gesichtschirurgie Universitätsmedizin Greifswald, Greifswald; ^3^ Gemeinschaftspraxis Dres. Peter/ Seeber/ Altheide/ von Georg, Hamburg

**Keywords:** Komplikationen, Nebenwirkungen, Laser, Energie‐basierte Systeme, Hyperpigmentierung, Hypopigmentierung, Patientensicherheit, complications, adverse events, laser, energy‐based devices, hyperpigmentation, hypopigmentation, scarring, patient safety

## Abstract

Laser‐ und energiebasierte Geräte (EBDs) sind in der Dermatologie fest etabliert und werden sowohl für medizinische als auch ästhetische Zwecke eingesetzt. Obwohl moderne Systeme über fortschrittliche Sicherheitsmechanismen wie integrierte Kühlsysteme und Echtzeit‐Monitoring verfügen, sind diese Verfahren nicht risikofrei. Komplikationen können durch eine unpassende Gerätewahl, suboptimale Behandlungsparameter, unzureichende Patientenevaluation oder mangelnde Erfahrung der Behandler entstehen. Dieser CME‐Artikel gibt einen Überblick über das Spektrum möglicher Nebenwirkungen bei dermatologischen Laser‐ und EBD‐Behandlungen, von erwartbaren kurzfristigen Reaktionen bis hin zu Spätkomplikationen wie Pigmentveränderungen, Narbenbildung und okulären Verletzungen. Präventions‐ und Behandlungsstrategien werden besprochen, mit besonderem Augenmerk auf Patientenselektion, Behandlungsplanung und Nachsorge. Zudem werden regulatorische Rahmenbedingungen in Deutschland, wie die Verordnung zum Schutz vor schädlichen Wirkungen nichtionisierender Strahlung bei der Anwendung am Menschen (NiSV), sowie Initiativen wie das Komplikationsregister der Deutschen Dermatologischen Lasergesellschaft hervorgehoben, die zur weiteren Verbesserung der Patientensicherheit beitragen sollen. Ein umfassendes Verständnis der möglichen Risiken und ihrer Vermeidung ist entscheidend, um die Patientensicherheit zu gewährleisten und klinische Ergebnisse in der Laserdermatologie zu optimieren.

## EINLEITUNG

Die Einführung von Lasern und Energie‐basierten Geräten (energy‐based devices, EBDs) in die dermatologische Praxis sowie in weitere medizinische Disziplinen hat die Behandlung sowohl medizinischer als auch ästhetischer Indikationen grundlegend verändert. In den vergangenen Jahrzehnten wurde das therapeutische Spektrum deutlich erweitert: Neben klassischen chirurgischen und pharmakologischen Verfahren stehen heute zahlreiche Technologien zur Verfügung, darunter Farbstofflaser, ablative und nicht‐ablative fraktionierte Laser, Nano‐ und Pikosekundenlaser, intensives gepulstes Licht (IPL), Radiofrequenz sowie weitere Energie‐basierte Systeme.^1^, ^2^ Diese Entwicklungen haben nicht nur die therapeutischen Möglichkeiten erheblich erweitert, sondern auch die Erwartungen der Patienten an minimalinvasive Verfahren mit verkürzten Ausfallzeiten und verbesserten ästhetischen Ergebnissen gesteigert.

Eine zentrale Grundlage der modernen Laserdermatologie bildet das Prinzip der selektiven Photothermolyse, das 1983 von Anderson und Parrish beschrieben wurde.^3^ Dieses Konzept ermöglicht eine gezielte Zerstörung spezifischer Chromophoren – wie Hämoglobin, Melanin oder Wasser – unter weitgehender Schonung des umgebenden Gewebes und trägt damit wesentlich zur Sicherheit und Präzision Laser‐basierter Behandlungen bei.^3^ Ein entscheidender Parameter ist dabei die thermische Relaxationszeit (thermal relaxation time, TRT) der Zielstruktur, die die Zeitspanne beschreibt, in der 50% der aufgenommenen Wärme wieder abgegeben werden. Um thermische Schädigungen zu reduzieren, sollte die Pulsdauer des Lasers gleich oder kürzer als die TRT des jeweiligen Chromophors sein.^3^, ^4^

Aufbauend auf diesem physikalischen Grundprinzip verfügen moderne Laser‐ und EBD‐Systeme zusätzlich über fortschrittliche Sicherheitsmechanismen. Dazu zählen integrierte Kühlsysteme sowie Echtzeit‐Überwachungen von Temperatur und Impedanz, die während der Behandlung ein dynamisches Feedback liefern und das Risiko einer Überhitzung oder überschießenden Gewebeschädigung reduzieren sollen.^5^, ^6^, ^7^, ^8^

Trotz dieser technologischen Fortschritte lassen sich Nebenwirkungen und Komplikationen nicht vollständig vermeiden.^9^, ^10^ Art und Ausmaß unerwünschter Ereignisse hängen von einer Vielzahl von Faktoren ab, darunter die Wahl des Geräts, die Einstellung der Behandlungsparameter, patientenspezifische Risikofaktoren sowie die Erfahrung und Expertise des behandelnden Arztes. Während einige Komplikationen vorübergehend und selbstlimitierend sind, können andere schwerwiegender Natur sein und langfristige funktionelle oder ästhetische Beeinträchtigungen nach sich ziehen. Vor diesem Hintergrund fasst der vorliegende Artikel die aktuelle Literatur zusammen und ergänzt diese durch klinische Erfahrung, um Ätiologie, Klassifikation sowie Strategien zur Prävention von Komplikationen bei Laser‐ und EBD‐Behandlungen darzustellen.

## ÄTIOLOGIE UND RISIKOFAKTOREN

Komplikationen bei Laser‐ und EBD‐Behandlungen können durch patientenbezogene, eingriffsbezogene oder anwenderbezogene Faktoren entstehen. Die sorgfältige Berücksichtigung all dieser Aspekte ist entscheidend, um die Behandlungssicherheit zu erhöhen und bestmögliche Therapieergebnisse zu erzielen.

### Patientenbezogene Risikofaktoren

Unter den patientenbezogenen Faktoren kommt dem Fitzpatrick‐Hauttyp eine zentrale Bedeutung zu.^11^ Personen mit dunkleren Hauttypen weisen ein erhöhtes Risiko für Komplikationen auf, da der erhöhte Melaningehalt der Epidermis einen Teil der Laserenergie absorbiert, die eigentlich auf andere Chromophore wie Wasser, Hämoglobin oder melaninreiche Zielstrukturen gerichtet ist. Diese konkurrierende Absorption vermindert die Selektivität der Behandlung und begünstigt pigmentäre Veränderungen wie postinflammatorische Hyperpigmentierungen oder Hypopigmentierungen.^12^, ^13^

Auch Begleiterkrankungen und Medikationen beeinflussen das individuelle Risikoprofil. Patienten mit Photosensibilität oder unter Einnahme photosensibilisierender Medikamente, wie beispielsweise Tetrazykline oder Amiodaron, können ausgeprägte entzündliche Reaktionen entwickeln.^14^ Bei diesen Patienten sollte, sofern möglich, abhängig vom geplanten Verfahren entweder eine Testbehandlung (Testspots) durchgeführt oder eine vorübergehende Unterbrechung der Medikation erwogen werden. Die Leitlinien der British Medical Laser Association aus dem Jahr 2009 stufen nicht zwingend notwendige ästhetische Laserbehandlungen bei Patienten mit systemischer oder lokaler Photosensibilität als kontraindiziert ein.^15^ Bis zum Jahr 2014 lagen jedoch keine publizierten Berichte über unerwünschte Wirkungen von Laserbehandlungen bei Patienten unter photosensibilisierender Medikation vor.^16^ Nach aktuellem Kenntnisstand existieren weiterhin keine spezifischen Empfehlungen hinsichtlich der Dauer einer Pausierung der Medikamente oder eine standardisierte Liste relevanter Arzneimittel.

Ebenso stellt das Vorliegen aktiver bakterieller, viraler oder mykotischer Infektionen einen relevanten Risikofaktor dar, da Laser‐ oder EBD‐Behandlungen zu einer lokalen Ausbreitung oder Exazerbation der Infektion führen können. Insbesondere die Reaktivierung einer Herpes‐simplex‐Infektion ist eine bekannte Komplikation, vor allem im Zusammenhang mit ablativen Lasertherapien.^17^, ^18^

Ein weiterer entscheidender Aspekt ist die UV‐Exposition. Eine Sonnenexposition, die kürzlich vor oder nach dem Eingriff stattgefunden hat, erhöht das Risiko für Pigmentveränderungen und thermische Hautschäden.^19^ Aus diesem Grund ist eine umfassende Patientenaufklärung essenziell. Diese sollte einen konsequenten UV‐Schutz über einen Zeitraum von vier Wochen vor sowie vier Wochen nach der Behandlung beinhalten. Empfohlen wird die Anwendung eines Sonnenschutzmittels mit einem Lichtschutzfaktor von mindestens 50 sowie zusätzlicher Schutz durch geeignete Kleidung. Ist dies, insbesondere in den Sommermonaten, nicht zuverlässig umsetzbar, sollte eine Behandlungspause in Erwägung gezogen werden. Zudem sollte dekorative Kosmetik im Behandlungsareal vor der Therapie vermieden oder vollständig entfernt werden.

Von gleicher Bedeutung ist eine Anleitung zur postinterventionellen Wundpflege und Infektionsprophylaxe.^20^, ^21^ Diese präventiven Maßnahmen tragen nicht nur maßgeblich zur Sicherheit der Behandlung bei, sondern helfen auch, realistische Erwartungen zu vermitteln und die Patientenzufriedenheit zu erhöhen.

Zu den patientenbezogenen Risikofaktoren zählen vor allem der Fitzpatrick‐Hauttyp und die UV‐Belastung zu den besonders entscheidenden Faktoren.

### Eingriffs‐ und anwenderspezifische Risikofaktoren

Neben patientenbezogenen Aspekten spielen eingriffs‐ und anwenderspezifische Risikofaktoren eine zentrale Rolle und sind für einen Großteil der Laser‐ und EBD‐assoziierten Komplikationen verantwortlich.^22^ Die Auswahl des geeigneten Systems sowie die Festlegung der Behandlungsparameter müssen sorgfältig an die jeweilige Indikation und den Hauttyp des Patienten angepasst werden. Der Einsatz ungeeigneter Systeme oder nicht optimal gewählter Einstellungen erhöht das Komplikationsrisiko erheblich. Zu hohe Energiedichten, ungeeignete Pulsdauern oder eine unzureichende Kühlung können epidermale und dermale Schädigungen verursachen, einschließlich Verbrennungen, Narbenbildung und Pigmentstörungen. Ablative *Resurfacing*‐Verfahren können zwar sehr wirksam sein, gehen jedoch grundsätzlich mit einem höheren Risikoprofil einher als nicht‐ablative Methoden. Ihre Sicherheit hängt maßgeblich von einer präzisen Parameterwahl und einer korrekten Durchführung ab. Nicht‐ablative Verfahren weisen in der Regel ein günstigeres Sicherheitsprofil auf, insbesondere bei dunkleren Hauttypen. Dennoch können auch hier unerwünschte Ereignisse auftreten, wenn sie ohne ausreichende Vorsicht angewendet werden.

Neben patientenbezogenen Aspekten spielen eingriffs‐ und anwenderspezifische Risikofaktoren eine zentrale Rolle und sind für einen Großteil der Laser‐ und EBD‐assoziierten Komplikationen verantwortlich.^22^ Die Auswahl des geeigneten Systems sowie die Festlegung der Behandlungsparameter müssen sorgfältig an die jeweilige Indikation und den Hauttyp des Patienten angepasst werden.

Unzureichende Ausbildung, begrenzte Erfahrung mit unterschiedlichen Hauttypen oder eine zu starke Orientierung an herstellerseitigen Standardeinstellungen können zu vermeidbaren Komplikationen führen. Eine kompetente Anwendung setzt daher eine sorgfältige Patientenselektion, realistische Aufklärung, eine präzise intraoperative Technik sowie eine erhöhte Aufmerksamkeit für frühe Warnzeichen einer Gewebeschädigung voraus, wie etwa unangemessene Schmerzen, ausgeprägtes Erythem, Blasenbildung, graue Verfärbungen oder Verbrennungen.

Eine kompetente Anwendung setzt daher eine sorgfältige Patientenselektion, realistische Aufklärung, eine präzise intraoperative Technik sowie eine erhöhte Aufmerksamkeit für frühe Warnzeichen einer Gewebeschädigung voraus, wie etwa unangemessene Schmerzen, ausgeprägtes Erythem, Blasenbildung, graue Verfärbungen oder Verbrennungen.

## ERWARTBARE PHYSIOLOGISCHE REAKTIONEN

Laser‐basierte dermatologische Verfahren führen häufig zu kurzfristigen Reaktionen, die direkte Folgen gezielter thermischer, photomechanischer oder photochemischer Wechselwirkungen mit dem Gewebe sind. Die häufigsten unmittelbaren Reaktionen umfassen Erythem, Ödem, Purpura und Krusten, die in der Regel gutartig und selbstlimitierend sind. Diese Reaktionen sind zu erwarten, typischerweise mild und selbstlimitierend und sollten daher eher als erwartbare Nebenwirkungen als echte Komplikationen betrachtet werden.

Laser‐basierte dermatologische Verfahren führen häufig zu kurzfristigen Reaktionen, die direkte Folgen gezielter thermischer, photomechanischer oder photochemischer Wechselwirkungen mit dem Gewebe sind.

Das Erythem ist eine der häufigsten Reaktionen nach Laser‐ und EBD‐Behandlungen. Schweregrad und Dauer hängen von den Geräteparametern ab, wie Wellenlänge, Energiedichte, Pulsdauer und Eindringtiefe sowie von patientenbezogenen Faktoren, wie z.B. Hautempfindlichkeit.^23^ Während das Erythem in der Regel innerhalb weniger Stunden bis Tage abklingt, kann seine Intensität durch intraoperative Kühlsysteme und geeignete Nachsorge gemildert werden.^5^, ^23^

Ödeme treten häufig zusammen mit Erythemen auf und spiegeln eine akute Entzündungsreaktion wider. Sie sind tendenziell ausgeprägter in Bereichen mit lockerem Bindegewebe, wie der periorbitalen Region, und können mehrere Tage anhalten.^23^ Unterstützende Maßnahmen, einschließlich kalten Kompressen, Kopfhochlagerung und in einigen Fällen entzündungshemmender Mittel wie topischer Steroide für wenige Tage nach der Behandlung, können helfen, Beschwerden zu lindern und die Rückbildung zu beschleunigen.

Eine Purpura entsteht, wenn vaskuläre Laser, insbesondere gepulste Farbstofflaser, fokale Kapillarrupturen hervorrufen. Im Kontext der Behandlung vaskulärer Läsionen stellt die Purpura oft ein gewünschter klinischer Endpunkt dar.^24^ Diese Makulae klingen normalerweise innerhalb von ein bis zwei Wochen spontan ab, ohne bleibende Folgen.

Krustenbildung und punktförmige Blutungen können nach ablativen Laserbehandlungen auftreten.^25^ Krusten sind in der Regel vorübergehend und lösen sich innerhalb weniger Tage bis Wochen auf, während die Reepithelisierung fortschreitet.^25^ Eine angemessene Wundversorgung, einschließlich sanfter Reinigung und Anwendung von pflegenden Topika, ist entscheidend, um Sekundärinfektionen zu vermeiden und eine optimale Heilung zu unterstützen.

Obwohl diese unmittelbaren Reaktionen vorübergehend und selten klinisch relevant sind, bleibt eine Patientenaufklärung vor der Behandlung essenziell. Eine klare Kommunikation über die zu erwartenden kurzfristigen Veränderungen sowie Anweisungen zur Nachsorge trägt dazu bei, Erwartungen zu steuern, Ängste zu reduzieren und die Einhaltung der Nachsorgeempfehlungen zu fördern.

## UNMITTELBARE KOMPLIKATIONEN

Unmittelbare Komplikationen nach Laser‐ und EBD‐Behandlungen treten typischerweise innerhalb von Stunden bis Tagen nach der Behandlung auf. Während die meisten vorübergehend und selbstlimitierend sind, ist in einigen Fällen ein Eingreifen erforderlich, um langfristige Folgen zu verhindern.

### Blasenbildung

Blasen können entstehen, wenn übermäßige Energie abgegeben oder eine unzureichende epidermale Kühlung angewendet wird.^26^ Sie spiegeln eine akute thermische Schädigung der Epidermis oder der dermo‐epidermalen Verbindung wider. Obwohl Blasen bei entsprechender Versorgung normalerweise ohne Narbenbildung abheilen, kann unangemessene Nachsorge oder eine Sekundärinfektion die Heilung verzögern und das Risiko postinflammatorischer Pigmentveränderungen erhöhen. Präventive Maßnahmen umfassen die sorgfältige Anpassung von Energiedichte und Pulsdauer an den Hauttyp der Patienten sowie die Verwendung effektiver Kühlsysteme.

### Infektiöse und entzündliche Reaktionen

Eine Schädigung der epidermalen Barriere begünstigt bakterielle oder virale Sekundärinfektionen.^27^ Die Reaktivierung des Herpes‐simplex‐Virus ist insbesondere nach ablativen Verfahren relevant. Für Patienten mit einer Vorgeschichte von Herpes labialis, die sich einer *Resurfacing*‐ oder anderen Hochrisikobehandlung unterziehen, wird eine prophylaktische antivirale Therapie empfohlen.^17^, ^18^ Nach unserem Kenntnisstand finden sich in der Literatur keine einheitlichen Empfehlungen zur Dosierung oder Dauer einer systemische Prophylaxe. In der Praxis wird üblicherweise eine niedrige Dosierung etwa 24 Stunden vor dem Eingriff begonnen und ein bis sieben Tage danach fortgeführt.^28^, ^29^ Strikte Hygienemaßnahmen und angemessene Wundversorgung minimieren zusätzlich das Infektionsrisiko.

Akneiforme Ausschläge können nach bestimmten Laser‐ und energiegestützten Verfahren auftreten, insbesondere wenn postinterventionell okklusive Verbände oder stark fettende Präparate verwendet werden.^30^ Follikuläre Okklusionen und lokale Entzündungsreaktionen tragen zu dieser Reaktion bei.^30^ Typischerweise sind die Läsionen selbstlimitierend, in einigen Fällen kann jedoch eine topische oder systemische Therapie erforderlich sein. Die Anpassung der Hautpflege nach der Behandlung und das Vermeiden stark okklusiver Produkte sind nützliche präventive Maßnahmen.

Während der Reepithelisierung nach ablativen Behandlungen können Milien entstehen.^31^ Diese kleinen, keratingefüllten Zysten sind gutartig, können jedoch kosmetische Bedenken bei Patienten hervorrufen. Eine sterile Exprimierung ist möglich. Sanfte Reinigung und nicht‐komedogene Präparate während der Heilungsphase helfen, ihre Entstehung zu reduzieren.

## SPÄTKOMPLIKATIONEN

Spätkomplikationen treten typischerweise Wochen bis Monate nach Laser‐ und EBD‐Behandlungen auf und können permanent werden, wenn sie nicht rechtzeitig erkannt und behandelt werden. Ein umfassendes Verständnis ihrer Risikofaktoren sowie evidenzbasierter Präventionsstrategien ist wesentlich, um die Patientensicherheit und Behandlungsergebnisse zu optimieren.

Spätkomplikationen treten typischerweise Wochen bis Monate nach Laser‐ und EBD‐Behandlungen auf und können permanent werden, wenn sie nicht rechtzeitig erkannt und behandelt werden.

### Verbrennungen

Verbrennungen stellen eine potenziell schwere Komplikation dar, die auftreten können, wenn eine zu hohe Energiedichte verwendet wurde oder Kühlmechanismen unzureichend waren. Ein frühes Warnzeichen kann eine Graufärbung der Haut direkt nach der Behandlung sein. Oberflächliche Verbrennungen können mit vorübergehenden Pigmentveränderungen abheilen, während tiefe dermale Verbrennungen das Risiko permanenter Narben oder struktureller Hautveränderungen bergen. Das Risiko ist erhöht bei Patienten mit kürzlich erfolgter UV‐Exposition, Skin of Color oder zugrunde liegenden Erkrankungen, die die Wundheilung beeinträchtigen. Prävention basiert auf individueller Auswahl der Behandlungsparameter und kontinuierlicher Überwachung während des Eingriffs. Tritt eine Verbrennung auf, ist eine sofortige Intervention erforderlich, beispielsweise ausreichende Kühlung, Wundversorgung, topische Kortikosteroide oder Antibiotika bei Verdacht auf eine Sekundärinfektion, um langfristige Folgen zu minimieren.

### Pigmentveränderungen

Hypo‐ und Hyperpigmentierungen gehören zu den häufigsten langfristigen Komplikationen, insbesondere bei Patienten mit dunkler Hautfarbe.^32^ Pigmentveränderungen entstehen oft durch übermäßige Energiedichte oder Pulsdauer, unzureichende Kühlung, ungeeignete Wellenlänge für den Hauttyp oder zu kurze Behandlungsintervalle.^33^ Strikter Lichtschutz, Kühlung, prä‐ und post‐interventionelle Externa sowie sorgfältige Anpassung der Parameter sind wichtige Strategien zur Prävention und Behandlung.

### Ulzerationen

**ABBILDUNG 1:**
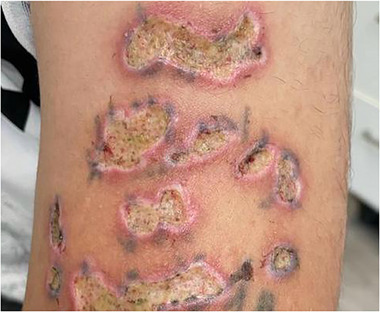
Patient mit Ulzerationen am Arm nach Laser‐Tattooentfernung. Mögliche, mitursächliche Fehlerquellen: Pulsdauer über der thermischen Relaxationszeit, hohe Energiedichte, nicht geeignetes System.

Ulzerationen können typischerweise durch übermäßige Energiedichten, längere Pulsdauern als die TRT des Zielchromophors oder unzureichende Kühlung verursacht werden. Sie treten auf, wenn Epidermis und Dermis über die regenerative Kapazität des Gewebes hinaus geschädigt werden, was zu offenen Wunden führen kann. Diese können das Risiko sekundärer Infektionen und verzögerter Heilung erhöhen (Abbildung 1). Frühe Anzeichen sind lokalisiertes Erythem, Blasenbildung oder anhaltende Schmerzen an der Behandlungsstelle. Eine sorgfältige Anpassung der Geräteparameter an Hauttyp und Indikation sowie eine gewissenhafte Nachsorge sind entscheidend, um das Risiko zu minimieren. Eine frühzeitige Erkennung und Behandlung, einschließlich Wundversorgung und Infektionsprävention, ist wesentlich, um langfristige Folgen wie Pigmentveränderungen und Narbenbildung zu verhindern.

### Narben

**ABBILDUNG 2:**
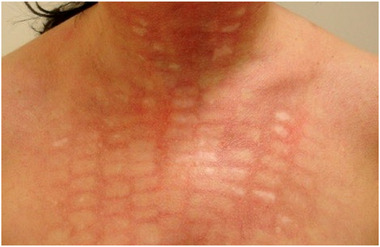
Patientin mit multiplen hypopigmentierten Narben am Dekollete und Hals nach Behandlung einer Erythrosis mit intensiv gepulstem Licht. Mögliche, mitursächliche Fehlerquelle: zu hohe Energiedichte.

Narben stellen eine der schwerwiegendsten Spätkomplikationen dar. Sie können durch tiefe dermale Verletzungen, sekundäre Infektionen oder eine verzögerte Wundheilung, insbesondere bei ablativen Verfahren, entstehen. Hypertrophe oder atrophe Narben sowie Keloide können auftreten und erhebliche kosmetische sowie psychologische Belastungen verursachen (Abbildung 2). Die Prävention basiert auf sorgfältiger Behandlungsplanung und angemessener Nachsorge. Etablierte Narben können eine multimodale Therapie erfordern, einschließlich Gefäßlaser, fraktionierter Laserbehandlungen oder Kortikosteroid‐Injektionen.

### Paradoxer Farbumschlag

Ein paradoxer Farbumschlag kann bei der Entfernung von Tätowierungen oder Permanent‐Make‐up beobachtet werden, insbesondere bei bestimmten Pigmenten wie Eisenoxid oder Titandioxid. Statt der Entfernung oxidiert das Pigment und dunkelt nach, was zu einem unerwünschten kosmetischen Ergebnis führen kann.^34^ Vor der Behandlung können Testflächen empfohlen werden. Sollte ein paradoxer Farbumschlag auftreten, sind oft nachfolgende Lasersitzungen wirksam, um das Ergebnis schrittweise zu verbessern.^35^, ^36^

### Paradoxe Hypertrichose

Eine paradoxe Haarstimulation wurde nach Laserepilationen beschrieben, insbesondere wenn subtherapeutische Energiedichten angewendet werden.^37^ Statt das Haarwachstum zu reduzieren, kann die Behandlung ruhende Follikel stimulieren, was zu einer erhöhten Haardichte im behandelten oder angrenzenden Bereich führt.^38^ Die Anpassung der Behandlungsparameter und eine sorgfältige Patientenauswahl sind entscheidende präventive Maßnahmen.

### Unbeabsichtigte Haarentfernung

Laser‐ oder EBD‐Behandlungen, die nicht primär zur Haarentfernung vorgesehen sind, können dennoch eine unbeabsichtigte Photoepilation erzeugen, wenn ihre Wellenlängen auch die Haarfollikel beeinflussen. Ein typisches Beispiel ist der Einsatz von langgepulsten Neodym‐dotierten Yttrium‐Aluminium‐Granat‐(Nd:YAG)‐Lasern zur Behandlung vaskulärer Veränderungen, wie Rosazea‐assoziierte Teleangiektasien im Wangenbereich bei männlichen Patienten. Patienten sollten im Vorfeld über diese mögliche Nebenwirkung informiert werden. Ein weiteres Beispiel, wenn auch in der Regel weniger ausgeprägt, ist die Behandlung von Permanent‐Make‐up im Augenbrauen‐ oder Lidrandbereich mit Nd:YAG‐Lasern. Auch in diesen Fällen sollten Patienten vor der Behandlung über das mögliche Risiko eines unerwünschten Haarverlusts aufgeklärt werden.

### Komplikationen am Auge

Komplikationen am Auge sind selten, können jedoch potenziell schwerwiegend sein. Eine direkte oder reflektierte Einwirkung von Laser‐ oder IPL‐Systemen kann je nach verwendeter Wellenlänge und Energie zu Hornhautverbrennungen, Irisdeformationen, fehlender Miosis, Kataraktbildung oder Netzhautverletzungen führen.^39^, ^40^, ^41^ Selbst bei Behandlungen, die nicht direkt am Auge stattfinden, ist der konsequente Gebrauch eines Augenschutzes sowohl für Patienten als auch für das Personal obligat. Zu den vorbeugenden Maßnahmen zählen wellenlängenangepasste Schutzbrillen, intraokulare Metall‐Augenschalen bei periorbitalen Verfahren sowie die strikte Einhaltung der Sicherheitsprotokolle.^42^ Augenschalen aus Kunststoff oder nicht zertifizierte Augenschutzmaßnahmen sind zu vermeiden.^42^ Bei Verdacht auf eine okuläre Verletzung ist eine sofortige ophthalmologische Untersuchung erforderlich.

Eine direkte oder reflektierte Einwirkung von Laser‐ oder IPL‐Systemen kann je nach verwendeter Wellenlänge und Energie zu Hornhautverbrennungen, Irisdeformationen, fehlender Miosis, Kataraktbildung oder Netzhautverletzungen führen.^39^, ^40^, ^41^

Tabelle 1 fasst die erwarteten Nebenwirkungen sowie die unmittelbaren und späten Komplikationen nach Laser‐ und EBD‐Behandlungen zusammen.

**TABELLE 1:** Zusammenfassung der erwartbaren physiologischen Reaktionen sowie der unmittelbaren und Spätkomplikationen nach Behandlungen mit Laser‐ und Energie‐basierten Geräten, einschließlich deren Prävention und Behandlung.

Kategorie	Subkategorie	Details	Ätiologie/ Risikofaktoren	Prävention / Management
**Erwartbare physiologische Reaktionen**	Erythem	Rötung infolge thermischer/ photomechanischer Effekte	Abhängig von Wellenlänge, Fluenz, Pulsdauer und Hautempfindlichkeit	Kühlung, topische oder orale Kortikosteroide
	Ödem	Schwellung in den behandelten Arealen	Häufig begleitet von Erythem, verstärkt in Arealen mit lockerem Bindegewebe, z. B. periorbital	Kühlung, Hochlagerung, bei Bedarf entzündungshemmende Medikamente
	Purpura	Kapillarrupturen durch vaskulär zielgerichtete Laser, z. B. bei Feuermalen	Oft gewünschter klinischer Endpunkt bei vaskulären Läsionen	Selbstlimitierend; Rückbildung typischerweise innerhalb von 1–2 Wochen
	Krusten	Epidermale Schädigung durch ablative bzw. weniger ablative Laser	Teil des Heilungsprozesses	Sanfte Reinigung, pflegende Präparate, Infektionsprophylaxe
**Unmittelbare Komplikationen**	Blasen	Thermische Schädigung der Epidermis bzw. der dermo‐epidermalen Übergangszone	Übermäßige Energieabgabe, unzureichende Kühlung	Anpassung von Energiedichte und Pulsdauer, effektive Kühlung, angemessene Nachsorge
	Infektionen/ Entzündungen	Bakterielle oder virale Infektionen, Herpesreaktivierung, akneiforme Eruptionen, Milien	Störung der epidermalen Barriere, okklusive Verbände	Prophylaktische antivirale Therapie, Hygienemaßnahmen, sanfte Reinigung, nicht‐komedogene Produkte
**Spätkompliationen**	Verbrennungen	Oberflächlich → vorübergehende Pigmentveränderungen; tief → Narbenbildung	Übermäßige Energie, unzureichende Kühlung, dunklere Hauttypen, kürzliche Sonnenexposition	Individualisierte Parameterwahl, intra‐prozedurales Monitoring, Kühlung, rasche Wundversorgung
	Pigmentveränderungen	Hyperpigmentierung oder Hypopigmentierung	Hohe Energiedichte, lange Pulsdauer, unzureichende Kühlung, dunklere Fitzpatrick‐Hauttypen	Strikter Lichtschutz, sorgfältige Auswahl von System und Parametern, topische Kortikosteroide
	Ulzerationen	Zerstörung der epidermalen und dermalen Schichten	Hohe Energiedichte, lange Pulsdauer, unzureichende Kühlung	Sorgfältige Behandlungsplanung, kontinuierliche Neubewertung der Parameter während der Behandlung, adäquate Wundversorgung
	Vernarbungen	Hypertrophe, atrophe Narben oder Keloide	Tiefe dermale Schädigung, sekundäre Infektion, Überbehandlung	Sorgfältige Planung, angemessene Nachsorge, multimodale Therapie bei etablierten Narben
	Paradoxer Farbumschlag	Farbumschlag von Tätowierungen oder Permanent‐Make‐up nach Laserbehandlung	Oxidation von Tätowierungspigmenten (Eisenoxid, Titandioxid)	Testareale, bei Auftreten weitere Lasersitzungen
	Paradoxe Haarstimulation	Verstärktes Haarwachstum nach Laser‐Haarentfernung	Kann infolge subtherapeutischer Energiedichten auftreten	Korrekte Wahl der Energiedichte, sorgfältige Patientenselektion
	Unbeabsichtigte Haarentfernung	Epilationseffekt bei nicht zur Photoepilation vorgesehenen Behandlungen	Haarwachstum im Behandlungsareal bei Laserbehandlungen mit Wellenlängen, die eine Epilation induzieren können	Patientenaufklärung und/oder Vermeidung behaarter Areale
	Komplikationen am Auge	Hornhautverbrennungen, Irisdeformationen, fehlende Miosis, Kataraktbildung, Netzhautverletzungen	Direkte oder reflektierte Exposition, periorbitale Eingriffe	Zertifizierte Schutzbrillen oder intraokulare Augenschilde, strikte Sicherheitsprotokolle, augenärztliche Untersuchung bei Verdacht auf Verletzung

Selbst bei Behandlungen, die nicht direkt am Auge stattfinden, ist der konsequente Gebrauch eines Augenschutzes sowohl für Patienten als auch für das Personal notwendig.

## QUALITÄTSSICHERUNG

Die Gewährleistung der Patientensicherheit und die Minimierung von Komplikationen bei dermatologischen Laser‐ und EBD‐Verfahren erfordert einen strukturierten Ansatz zur Qualitätssicherung und Prävention.

### Rechtliche Anforderungen

Die NiSV (Verordnung zum Schutz vor schädlichen Wirkungen nichtionisierender Strahlung bei der Anwendung am Menschen), die 2022 in Deutschland in Kraft trat, legt gesetzlich verbindliche Anforderungen für die sichere Anwendung von Lasern und anderen nichtionisierenden EBDs in der ästhetischen Medizin fest.^43^ Ihr Hauptziel ist die Erhöhung der Patientensicherheit und die Standardisierung von Qualitätssicherungsmaßnahmen für alle Anwender. Nach der NiSV dürfen nur Personen mit entsprechender ärztlicher Ausbildung Hochrisikogeräte wie Laser und EBDs bedienen.^43^ Ärzte müssen zertifizierte Schulungen absolvieren, die theoretische und praktische Module umfassen, um ihre Kompetenz in Gerätebedienung, Patientenauswahl und Komplikationsmanagement nachzuweisen.^43^ Nicht‐ärztliches Personal ist in der Nutzung dieser Geräte eingeschränkt und muss strenge Aufsichts‐ und Anwendungsregelungen einhalten.^43^ Diese Vorgaben stellen sicher, dass nur ausreichend geschultes Personal potenziell risikobehaftete Verfahren durchführt, in Übereinstimmung mit den *Best‐Practice*‐Prinzipien der dermatologischen Lasermedizin.

Die NiSV (Verordnung zum Schutz vor schädlichen Wirkungen nichtionisierender Strahlung bei der Anwendung am Menschen), die 2022 in Deutschland in Kraft trat, legt gesetzlich verbindliche Anforderungen für die sichere Anwendung von Lasern und anderen nichtionisierenden EBDs in der ästhetischen Medizin fest.^43^

Darüber hinaus dürfen nach der NiSV nur approbierte Ärzte Behandlungen durchführen, die die Schädigung der epidermalen Barriere beinhalten, wie zum Beispiel. Tätowierungsentfernung, ablative Laserbehandlungen und vergleichbare Therapien, sofern sie Dermatologen, Plastische Chirurgen oder eine spezielle NiSV‐Zertifizierung für solche Verfahren haben.^43^

Die Verordnung schreibt außerdem eine detaillierte Dokumentation aller Verfahren vor, einschließlich Patientenaufklärung, Behandlungsparametern und auftretender Nebenwirkungen.^43^ Strukturierte Aufzeichnungen ermöglichen die kontinuierliche Qualitätssicherung und die Nachverfolgung von Komplikationen sowohl für interne als auch externe Überprüfungen. Zusätzlich verlangt die NiSV regelmäßige Gerätekontrollen, Wartung und Kalibrierung, um eine sichere und gleichmäßige Energieabgabe zu gewährleisten. Durch die Durchsetzung standardisierter Sicherheitsprüfungen und die Einhaltung der Herstelleranweisungen wird das Risiko gerätebedingter Komplikationen reduziert. Insgesamt soll die NiSV die Qualitätssicherung in der Laser‐ und EBD‐Anwendung fördern.

Durch die verpflichtende ärztliche Ausbildung, gründliche Dokumentation, Gerätesicherheitsmaßnahmen und gesetzliche Aufsicht bietet sie einen Rahmen zur Prävention von Komplikationen und zur Sicherstellung hoher Standards in der Patientenversorgung.

### Umfassende Ausbildung und Kompetenz

Eine entscheidende Voraussetzung für die sichere Anwendung von Lasern ist eine fundierte ärztliche Ausbildung. Programme wie der Master of Science in Differenzierter Ästhetischer Laser‐ und Plasmamedizin (DALM) bieten strukturierte, evidenzbasierte Lehrpläne. Diese stellen sicher, dass Ärzte fundierte Kenntnisse in Laserphysik, gerätespezifischen Parametern, Indikationen, Kontraindikationen und im Umgang mit Komplikationen erwerben.^44^, ^45^ Die Ausbildung sollte den Schwerpunkt auf praktische Erfahrungen unter fachkundiger Anleitung legen. Wichtig ist, dass der Einsatz medizinischer Laser und EBDs strikt im Verantwortungsbereich qualifizierter Ärzte verbleibt.

Eine entscheidende Voraussetzung für die sichere Anwendung von Lasern ist eine fundierte ärztliche Ausbildung. Programme wie der Master of Science in Differenzierter Ästhetischer Laser‐ und Plasmamedizin (DALM) bieten strukturierte, evidenzbasierte Lehrpläne. Diese stellen sicher, dass Ärztinnen und Ärzte fundierte Kenntnisse in Laserphysik, gerätespezifischen Parametern, Indikationen, Kontraindikationen und im Umgang mit Komplikationen erwerben.^44^, ^45^

### Systematische Überwachung und Komplikationsregister

Ein weiterer Grundpfeiler der Qualitätssicherung ist die systematische Erfassung unerwünschter Ereignisse. Die Einrichtung und Pflege von Registern für Laser‐ und EBD‐assoziierte Komplikationen ermöglicht kontinuierliches Lernen und erleichtert die Identifikation von Risikomustern über Geräte und Behandlungssituationen hinweg. Solche Register, wie das Komplikationsregister der Deutschen Dermatologischen Lasergesellschaft (DDL), erlauben es Ärzten, ihre Behandlungsergebnisse mit nationalen oder internationalen Daten zu vergleichen, was die kontinuierliche Verbesserung unterstützt und die Patientensicherheit erhöht. Darüber hinaus können Registerdaten zur Entwicklung zukünftiger Leitlinien und Ausbildungscurricula beitragen und so einen Rückkopplungsmechanismus zwischen klinischer Praxis und medizinischer Ausbildung schaffen.

## SCHLUSSFOLGERUNG

Laser und EBDs stellen einen Grundpfeiler der modernen Dermatologie dar und bieten ein breites Spektrum therapeutischer Möglichkeiten für verschiedene medizinische und ästhetische Indikationen. Ihr sicherer und effektiver Einsatz setzt jedoch ein fundiertes Verständnis der zugrunde liegenden Risikofaktoren, der zu erwartenden physiologischen Reaktionen sowie potenzieller Komplikationen voraus. Während die meisten Nebenwirkungen vorübergehend und selbstlimitierend sind, verdeutlichen mögliche Langzeitfolgen wie Pigmentveränderungen, Narbenbildung oder Verletzungen am Auge die Bedeutung von Prävention, frühzeitiger Erkennung und evidenzbasierter Behandlung.

Rechtliche Rahmenbedingungen wie die NiSV, strukturierte ärztliche Weiterbildung, eine sorgfältige Dokumentation sowie Komplikationsregister spielen eine zentrale Rolle bei der Standardisierung der Praxis und der Gewährleistung der Patientensicherheit. Die Kombination aus moderner Technologie, qualitätssichernden Maßnahmen und professioneller Expertise ist entscheidend, um Risiken zu minimieren, Behandlungsergebnisse zu optimieren und hohe Standards in der dermatologischen Versorgung aufrechtzuerhalten. Zukünftige Entwicklungen sollten zudem eine stärkere Einbindung aufsichtsführender Behörden berücksichtigen, um Sicherheit und Verantwortlichkeit weiter zu verbessern.

## DANKSAGUNG

Open access Veröffentlichung ermöglicht und organisiert durch Projekt DEAL.

## INTERESSENSKONFLIKT

LN hat Vortragshonorare von Cynosure Lutronic® erhalten. SH ist wissenschaftlicher Leiter des Postgraduiertenprogramms Master of Science in Differentierte Ästhetische Laser‐ und Plasmamedicine (DALM). NS ist Präsident der Deutschen Dermatologischen Lasergesellschaft (DDL). WK und SWS haben keine Angaben.

## CME Questions – Lernerfolgskontrolle

1. Was beschreibt die Theorie der selektiven Photothermolyse?
  - Die kontinuierliche Abgabe von Laserenergie unabhängig von Absorptionscharakteristika der Haut.
  - Die gezielte Zerstörung spezifischer Zielstrukturen (Chromophore) im Gewebe durch selektive Absorption von Laserenergie unter Schonung des umliegenden Gewebes.
  - Die unspezifische Erwärmung aller Gewebeschichten durch breitbandiges Licht, die zu einer globalen Thermoschädigung führt.
  - Die Erhöhung der Kollagenproduktion durch subtherapeutische Wärmeeinwirkung ohne direkte Gewebsdestruktion.
  - Die vollständige Abtragung der Epidermis und Dermis mit anschließender Reepithelisierung.
2. Welche Aussage ist richtig?
  - Die Parameter der Gerätehersteller können für jeden Patienten einfach übernommen werden.
  - Höhere Fluenzwerte sind grundsätzlich sicherer, da sie schneller wirken.
  - Die Parameter sollten je nach Indikation und Patienten‐Charakteristika angepasst werden.
  - Patientensicherheit hängt ausschließlich von der Gerätequalität ab, nicht vom Anwender.
  - Eine Anpassung der Pulsdauer ist nicht notwendig, solange das Gerät CE‐zertifiziert ist.
3. Was sind nicht zu erwartende Reaktionen der Haut nach einer Behandlung mit einem Laser oder Energie‐basierten System?
  - Erythem
  - Schwellung
  - Erhöhung der Leberwerte
  - Purpura
  - Krusten
4. Welche Aussage stimmt?
  - Man kann einfach über infizierte Areale lasern, da die Laserenergie die Keime abtötet.
  - In bestimmten Fällen ist eine präventive Herpes‐Therapie empfohlen.
  - Aktive bakterielle, virale oder mykotische Infektionen an der zu behandelnden Stelle stellen keine Kontraindikation für eine Laserbehandlung dar.
  - Hygienestandards und sorgfältige Wundpflege sind für die Infektionsprophylaxe nicht notwendig.
  - Nach ablativen Laserbehandlungen besteht kein Risiko einer Infektion, da die Hautbarriere intakt bleibt.
5. Langzeitkomplikationen nach Laser‐ und EBD‐Behandlungen können …
  - immer vollständig ausgeschlossen werden.
  - unter anderem Narbenbildung und Keloide umfassen.
  - ausschließlich bei Patienten mit dunklem Hauttyp nach Fitzpatrick auftreten.
  - durch routinemäßige Antibiotikagabe sicher verhindert werden.
  - ohne klinische Bedeutung sein und bedürfen keiner weiteren Behandlung.
6. Welche Aussage ist falsch?
  - Während einer Laserbehandlung müssen sowohl der Patient als auch der Behandler einen suffizienten Augenschutz tragen.
  - Im Rahmen von Laserbehandlungen können schwarze Augenschalen aus Kunststoff zum Schutz der Augen genutzt werden.
  - Der Behandler kann auf Augenschutz verzichten, wenn er den Laser nicht direkt in Richtung seiner Augen richtet.
  - Der Augenschutz muss für die jeweilige Laserwellenlänge geeignet sein.
  - Bei periokulären Behandlungen können Metall‐Augenschalen zum Einsatz kommen
7. Was reguliert die Nicht‐Ionisierende Strahlquellen‐Verordnung (NiSV)?
  - Die Anwendung von Laser‐ und anderen nichtionisierenden Strahlungsquellen zu kosmetischen Zwecken.
  - Den Arbeitsschutz beim Umgang mit ionisierender Strahlung (z. B. Röntgen, Computer‐Tomographie).
  - Die Herstellung und Zulassung von medizinischen Geräten mit Laser‐ oder Ultraschalltechnologie.
  - Die Strahlendosisgrenzwerte für radioaktive Substanzen in der Nuklearmedizin.
  - Die Vergütung von Laser‐ und Strahlentherapien durch gesetzliche Krankenkassen.
8. Wer darf laut der Nicht‐Ionisierenden Strahlquellen‐Verordnung (NiSV) eine Behandlung mit Lasersystemen und Energie‐basierten Geräten durchführen?
  - Jede kosmetische Fachkraft nach einer einwöchigen Geräteeinweisung durch den Hersteller.
  - Heilpraktikerinnen und Heilpraktiker ohne zusätzliche Qualifikation.
  - Alle Personen, die eine NiSV‐Fachkundeprüfung im Bereich Haut absolviert haben, unabhängig von der Indikation.
  - Nur approbierte Ärztinnen und Ärzte, wenn es sich um Therapien handelt, die die Epidermisbarriere schädigen (z. B. Tattooentfernung, ablative Therapien, etc.), sofern sie Dermatologen oder plastische Chirurgen sind oder die entsprechende NiSV‐Fachkunde erworben haben.
  - Kosmetikerinnen und Kosmetiker, sofern sie eine zweijährige Berufserfahrung nachweisen können.
9. Was beinhaltet die Nicht‐Ionisierende Strahlquellen‐Verordnung nicht?
  - Regelungen zur Anwendung von Lasern, intensiven gepulsten Lichtquellen, Ultraschall und Hochfrequenzgeräten zu nichtmedizinischen Zwecken.
  - Anforderungen an die Fachkunde und regelmäßige Fortbildung des anwendenden Personals.
  - Vorgaben zur Aufklärung, Dokumentation und zum Schutz der behandelten Personen.
  - Bestimmungen zur Herstellung und zum Inverkehrbringen von medizinischen Geräten.
  - Schutzmaßnahmen vor gesundheitlichen Risiken bei kosmetischen Laser‐ und Energieanwendungen.
10. Welchen Nutzen hat der DALM‐Studiengang (Master of Science in Differentiated Aesthetic Laser and Medicine)?
  - Er vermittelt eine strukturierte, evidenzbasierte Ausbildung in Laser‐ und energiebasierten Verfahren, einschließlich Indikationen, Kontraindikationen und Komplikationsmanagement.
  - Er berechtigt automatisch zur Anwendung aller kosmetischen Verfahren ohne zusätzliche Fachkunde.
  - Er ersetzt die gesetzlich vorgeschriebene NiSV‐Fachkunde vollständig.
  - Er dient ausschließlich der Geräteeinweisung durch Herstellerfirmen.
  - Er ist ausschließlich für Kosmetiker konzipiert.

Liebe Leserinnen und Leser, der Einsendeschluss an die DDA für diese Ausgabe ist der 29. Mai 2026.

Die richtige Lösung zum Thema Sklerosierende Erkrankungen der Haut in Heft 10/2025 ist: 1a, 2e, 3c, 4b, 5e, 6d, 7b, 8c, 9a, 10c

Bitte verwenden Sie für Ihre Einsendung das aktuelle Formblatt auf der folgenden Seite oder aber geben Sie Ihre Lösung online unter http://jddg.akademie-dda. de ein.

## References

[1] Raulin C , Karsai S . Laser and IPL technology in dermatology and aesthetic medicine. 2011;10:3‐40.

[2] Anderson RR . Lasers in dermatology—a critical update. J Dermatol. 2000;27(11):700‐705. 11138535 10.1111/j.1346-8138.2000.tb02262.x

[3] Anderson RR , Parrish JA . Selective photothermolysis: precise microsurgery by selective absorption of pulsed radiation. Science. 1983;220(4596):524‐527. 6836297 10.1126/science.6836297

[4] Stuart Nelson J , Milner TE , Svaasand LO , Kimel S . Laser pulse duration must match the estimated thermal relaxation time for successful photothermolysis of blood vessels. Lasers Med Sci. 1995;10(1):9‐12.

[5] Zenzie H , Altshuler G , Smirnov M , Anderson R . Evaluation of cooling methods for laser dermatology. Lasers Surg Med. 2000;26(2):130‐144. 10685086 10.1002/(sici)1096-9101(2000)26:2<130::aid-lsm4>3.0.co;2-j

[6] Bodendorf MO , Grunewald S , Simon JC , Paasch U . Efficacy and cosmetic results of contact gel cooling of the skin during non‐ablative laser procedures. J Dtsch Dermatol Ges. 2008;6(8):647‐652. 18201219 10.1111/j.1610-0387.2008.06610.x

[7] Raulin C , Greve B , Hammes S . Cold air in laser therapy: first experiences with a new cooling system. Lasers Surg Med. 2000;27(5):404‐410 11126434 10.1002/1096-9101(2000)27:5<404::AID-LSM1001>3.0.CO;2-S

[8] Lack EB , Rachel JD , D'Andrea L , Corres J . Relationship of energy settings and impedance in different anatomic areas using a radiofrequency device. Dermatol Surg. 2005;31(12):1668‐1670. 16336885 10.2310/6350.2005.31306

[9] Willey A , Anderson RR , Azpiazu JL , et al. Complications of laser dermatologic surgery. Lasers Surg Med. 2006;38(1):1‐15. 16444692 10.1002/lsm.20286

[10] Hammes S , Karsai S , Metelmann HR , et al. Treatment errors resulting from use of lasers and IPL by medical laypersons: results of a nationwide survey. J Dtsch Dermatol Ges. 2013;11(2):149‐156. 23194381 10.1111/j.1610-0387.2012.08042.x

[11] Soares I , Amaral IP , Correia MP , et al. Complications of dermatologic lasers in high Fitzpatrick phototypes and management: an updated narrative review. Lasers Med Sci. 2024;39(1):149. 38834924 10.1007/s10103-024-04100-4

[12] Shah S , Alster TS . Laser treatment of dark skin: an updated review. Am J Clin Dermatol. 2010;11(6):389‐397. 20866114 10.2165/11538940-000000000-00000

[13] Battle Jr EF , Soden Jr CE , editors. The use of lasers in darker skin types. Semin Cutan Med Surg; 2009: WB Saunders. 10.1016/j.sder.2009.04.003 19608064

[14] Kalka K , Merk H , Mukhtar H . Photodynamic therapy in dermatology. J Am Acad Dermatol. 2000;42(3):389‐413. 10688709 10.1016/s0190-9622(00)90209-3

[15] Association BML . Drugs and Lasers/IPLs. https://bmla.co.uk/drugs‐and‐laser‐ipls/2018 [Last accessed December 10, 2025].

[16] Kerstein RL , Lister T , Cole R . Laser therapy and photosensitive medication: a review of the evidence. Lasers Med Sci. 2014;29(4):1449‐1452. 24590242 10.1007/s10103-014-1553-0

[17] Alster TS , Nanni CA . Famciclovir prophylaxis of herpes simplex virus reactivation after laser skin resurfacing. Dermatol Surg. 1999;25(3):242‐246. 10193975 10.1046/j.1524-4725.1999.08197.x

[18] Beeson WH , Rachel JD . Valacyclovir prophylaxis for herpes simplex virus infection or infection recurrence following laser skin resurfacing. Dermatol Surg. 2002;28(4):331‐336. 11966791 10.1046/j.1524-4725.2002.01155.x

[19] Haedersdal M , Bech‐Thomsen N , Poulsen T , Wulf HC . Ultraviolet exposure influences laser‐induced wounds, scars, and hyperpigmentation: a murine study. Plast Reconstr Surg. 1998;101(5):1315‐1322. 9529218 10.1097/00006534-199804050-00024

[20] Duke D , Grevelink JM . Care before and after laser skin resurfacing. A survey and review of the literature. Dermatol Surg. 1998;24(2):201‐206. 9491114 10.1111/j.1524-4725.1998.tb04138.x

[21] Gold M , Andriessen A , Cohen JL , et al. Pre‐/postprocedure measures for laser/energy treatments: A survey. J Cosmet Dermatol. 2020;19(2):289‐295. 31840388 10.1111/jocd.13259

[22] Nanni CA , Alster TS . Complications of cutaneous laser surgery. A review. Dermatol Surg. 1998;24(2):209‐219. 9491115 10.1111/j.1524-4725.1998.tb04139.x

[23] Adamič M , Pavlović M , Troilius Rubin A , et al. Guidelines of care for vascular lasers and intense pulse light sources from the European Society for Laser Dermatology. J Eur Acad Dermatol Venereol. 2015;29(9):1661‐1678. 25931003 10.1111/jdv.13177

[24] Alam M , Dover JS , Arndt KA . Treatment of facial telangiectasia with variable‐pulse high‐fluence pulsed‐dye laser: comparison of efficacy with fluences immediately above and below the purpura threshold. Dermatol Surg. 2003;29(7):681‐684. 12828690 10.1046/j.1524-4725.2003.29181.x

[25] Alexiades‐Armenakas MR , Dover JS , Arndt KA . The spectrum of laser skin resurfacing: nonablative, fractional, and ablative laser resurfacing. J Am Acad Dermatol. 2008;58(5):719‐737. 18423256 10.1016/j.jaad.2008.01.003

[26] Zelickson Z , Schram S , Zelickson B . Complications in cosmetic laser surgery: a review of 494 Food and Drug Administration manufacturer and user facility device experience reports. Dermatol Surg. 2014;40(4):378‐382. 24826394 10.1111/dsu.12461

[27] Chiller K , Selkin BA , Murakawa GJ , editors. Skin microflora and bacterial infections of the skin. J Investig Dermatol Symp Proc; 2001: Elsevier. 10.1046/j.0022-202x.2001.00043.x 11924823

[28] Alster TS , Nanni CA . Famciclovir prophylaxis of herpes simplex virus reactivation after laser skin resurfacing. Dermatol Surg. 1999;25(3):242‐246. 10193975 10.1046/j.1524-4725.1999.08197.x

[29] Beeson WH , Rachel JD . Valacyclovir prophylaxis for herpes simplex virus infection or infection recurrence following laser skin resurfacing. Dermatol Surg. 2002;28(4):331‐336. 11966791 10.1046/j.1524-4725.2002.01155.x

[30] Mirza HN , Mirza FN , Khatri KA . Outcomes and adverse effects of ablative vs nonablative lasers for skin resurfacing: a systematic review of 1093 patients. Dermatol Ther. 2021;34(1):e14432. 33084193 10.1111/dth.14432

[31] Kaur J , Sharma S , Kaur T , Bassi R . Complications of fractional ablative carbon dioxide laser in various aesthetic procedures: a retrospective study. Int J Res Dermatol. 2019;5(4):664‐667.

[32] Kim YJ , Lee HS , Son SW , et al. Analysis of hyperpigmentation and hypopigmentation after Er: YAG laser skin resurfacing. Lasers Surg Med. 2005;36(1):47‐51. 15662626 10.1002/lsm.20120

[33] Graber EM , Tanzi EL , Alster TS . Side effects and complications of fractional laser photothermolysis: experience with 961 treatments. Dermatol Surg. 2008;34(3):301‐307. 18190541 10.1111/j.1524-4725.2007.34062.x

[34] Anderson RR , Geronemus R , Kilmer SL , et al. Cosmetic tattoo ink darkening: a complication of Q‐switched and pulsed‐laser treatment. Arch Dermatol. 1993;129(8):1010‐1014. 8352605 10.1001/archderm.129.8.1010

[35] Bae YSC , Alabdulrazzaq H , Brauer J , Geronemus R . Successful treatment of paradoxical darkening. Lasers Surg Med. 2016;48(5):471‐473. 26833886 10.1002/lsm.22482

[36] Kang S , Park S , Park J , et al. Paradoxical darkening following picosecond laser and successful treatment. Clin Exp Dermatol. 2021;46(6):1128‐1129. 33774841 10.1111/ced.14661

[37] Desai S , Mahmoud BH , Bhatia AC , Hamzavi IH . Paradoxical hypertrichosis after laser therapy: a review. Dermatol Surg. 2010;36(3):291‐298. 20100274 10.1111/j.1524-4725.2009.01433.x

[38] Town G , Bjerring P . Is paradoxical hair growth caused by low‐level radiant exposure by home‐use laser and intense pulsed light devices? J Cosmet Laser Ther. 2016;18(6):355‐362. 26983796 10.3109/14764172.2016.1157373

[39] Huang A , Phillips A , Adar T , Hui A . Ocular injury in cosmetic laser treatments of the face. J Clin Aesthet Dermatol. 2018;11(2):15. PMC5843357 29552271

[40] Juhasz M , Zachary C , Cohen JL . Ocular complications after laser or light‐based therapy—dangers dermatologists should know. Dermatol Surg. 2021;47(5):624‐629. 33731574 10.1097/DSS.0000000000002974

[41] Flegel L , Kherani F , Richer V . Review of eye injuries associated with dermatologic laser treatment. Dermatol Surg. 2022;48(5):545‐550. 35333214 10.1097/DSS.0000000000003427

[42] Nguyen L , Seeber N , Schneider SW , Herberger K . Thermal eye injuries from dermatologic laser treatments—an experimental study. Lasers Med Sci. 2023;38(1):110. 37086295 10.1007/s10103-023-03769-3 PMC10122618

[43] Verordnung zum Schutz vor schädlichen Wirkungen nichtionisierender Strahlung bei der Anwendung am Menschen (NiSV). BGBl. I Nr. 25/2020. 2020. https://www.gesetze‐im‐internet.de/nisv/BJNR218700018.html?utm_source=chatgpt.com. (Last accessed September 28, 2025).

[44] Metelmann H‐R , Hammes S , Hartwig K , et al. Safe and Effective Plasma Treatment by Structured Education. Comprehensive Clinical Plasma Medicine: Cold Physical Plasma for Medical Application: Springer; 2018.467‐472.

[45] Metelmann H‐R , Seebauer C , Hammes S . Postgraduate Education to Assure Quality Standards of Photonic Treatment. Energy for the Skin: Effects and Side‐Effects of Lasers, Flash Lamps and Other Sources of Energy. In: Energy for the Skin. Ed: Kautz G . Springer; 2022. 31‐36.

